# Microbiota effects on cancer: from risks to therapies

**DOI:** 10.18632/oncotarget.24681

**Published:** 2018-04-03

**Authors:** Domenica Rea, Giovanni Coppola, Giuseppe Palma, Antonio Barbieri, Antonio Luciano, Paola Del Prete, Sabrina Rossetti, Massimiliano Berretta, Gaetano Facchini, Sisto Perdonà, Maria Caterina Turco, Claudio Arra

**Affiliations:** ^1^ S.S.D Sperimentazione Animale, Istituto Nazionale Tumori, IRCCS, “Fondazione G. Pascale”, Naples, Italy; ^2^ Direzione Scientifica, Istituto Nazionale Tumori, IRCCS, “Fondazione G. Pascale”, Naples, Italy; ^3^ Division of Medical Oncology, Department of Uro-Gynaecological Oncology, Istituto Nazionale Tumori, IRCCS, “Fondazione G. Pascale”, Naples, Italy; ^4^ Department of Medical Oncology, CRO- Aviano, National Cancer Institute, Aviano, Italy; ^5^ Department of Urology, Istituto Nazionale Tumori, IRCCS, “Fondazione G. Pascale”, Naples, Italy; ^6^ Department of Medicine and Surgery, University of Salerno, Baronissi, Salerno, Italy

**Keywords:** gut microbiota, cancer, inflammation, probiotics, colon rectal cancer

## Abstract

Gut microbiota, a group of 10^14^ bacteria, eukaryotes and virus living in gastrointestinal tract, is crucial for many physiological processes in particular plays an important role in inflammatory and immune reactions. Several internal and external factors can influence this population, and shifts in their composition, have been demonstrated to contribute and affect different diseases. During dysbiosis several bacteria related to inflammation, one of the most necessary factors in carcinogenesis; it has been shown that some bacterial strains through deregulation of different signals/pathways may affect tumor development through the production of many factors. Gut microbiota might be considered as a holistic hub point for cancer development: direct and indirect involvements have been studying in several neoplasms such as colon rectal cancer, hepatocellular carcinoma and breast cancer. This review discuss over the evidence of crosstalk between gut microbiota and cancer, its ability to modulate chemotherapy, radiotherapy and immunotherapy, and the possibility that the intestinal microbial is a new target for therapeutic approaches to improve the prognosis and quality of life of cancer patients.

## INTRODUCTION

Digestive tracts of humans and animals contain a complex community of microorganisms called gut-microbiota which gets a deep mutualistic relationship with host mucosal epithelial cells and immune cells [1]. Those microorganisms are helpful in physiologic activities such as digestion, metabolism, epithelial homeostasis and development of gut associated lymphoid tissues; moreover they can metabolize bile acids and xenobiotics as well as synthesize Vitamin B and Vitamin K, while their antigens and their metabolic products can stimulate the production of cytokines against potential pathogens [1]. Human gut microbiota has the largest numbers of bacteria compared to other body’s districts. Up to 10^14^ bacteria and several archaea, eukaryotes and virus are present in gastrointestinal tract (GI) [2]. The composition of the gut microbiome changes with host’s age; and has its origin from placenta, amniotic fluid, umbilical cord blood, and meconium starting colonization of the fetus in utero. The placental microbiota can affect the growth and survival of the fetus and various postnatal pathologies. Preterm birth may have long-term consequences for the development of immune-mediated diseases (bronchial asthma, atopic dermatitis) [3, 4]. During the birth there is a vertical microbiome transmission, the newborns are exposed to vaginal microbes among the most frequent we mention: *Lactobacillus* and *Prevotella spp.,* while the babies born via C-section are exposed to skin microbes *Staphylococcus*, *Corynebacterium*, and *Propionibacterium spp.;* subsequently the intake of breast milk or formula milk influence the colonization process in the newborn. In the first years of baby born (2-3 years); the introduction of solid food contributed to increase the complexity of immature microbiome, puberty-associated influx of sex hormones introduces features related to gender specificity of the existing microbiome.

This process of microbiome maturation takes place in parallel with development of host organs, including the intestine, which elongates with age, providing additional niches for the microbiome to expand in number and diversity. After birth, about 100 bacterial species colonize the intestine; during weaning and growth of humans, the number and diversity of them increases to about 10^3^ bacterial species in adulthood; but their composition is constantly evolving [2–5].

**Figure 1:**
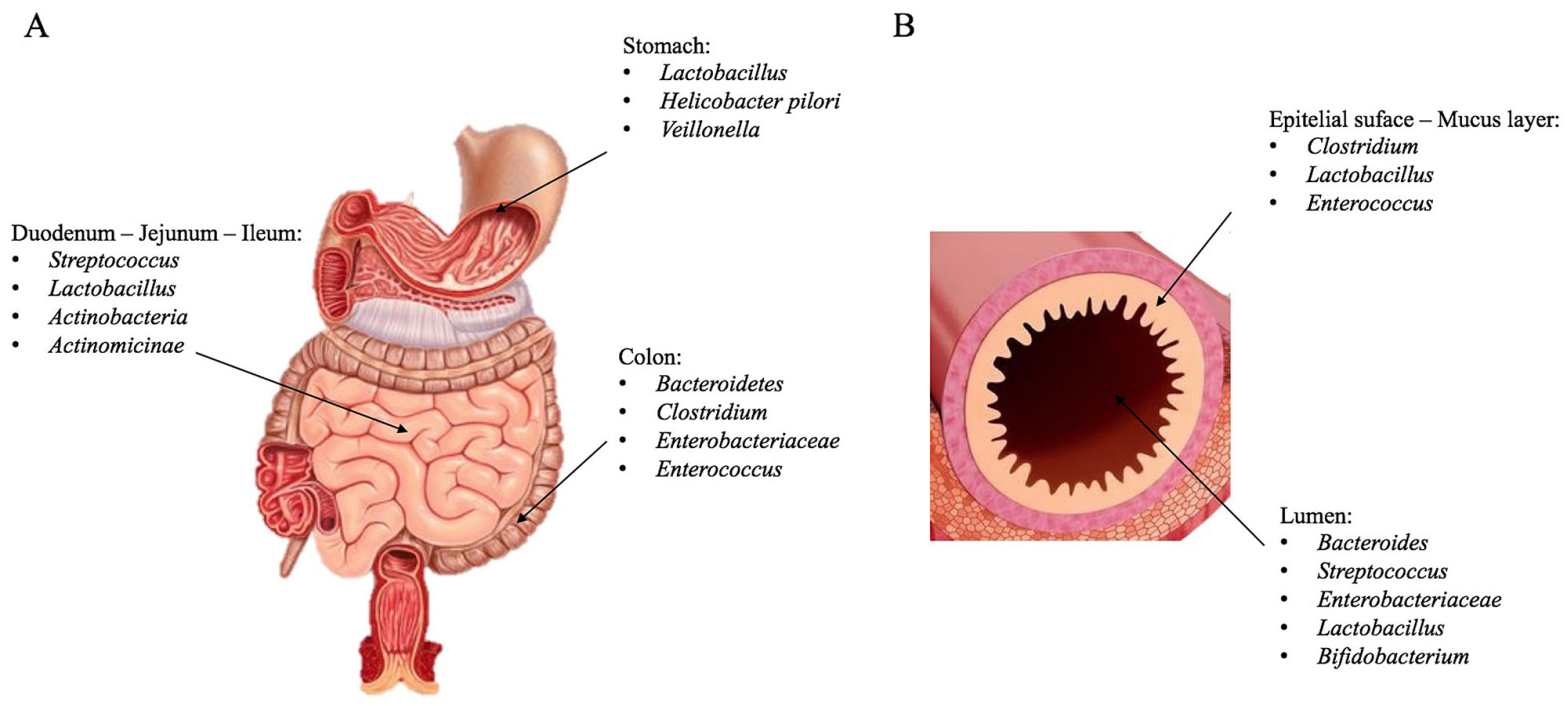
Microbiota composition Representative image of microbiota species in the GI **(A)**, in particular at the level of the duodenum-jejunum-ileum, stomach and colon. **(B)** Description of the species present in the intestinal lumen and in epithelial surface-mucus layer.

Several internal and external factors can influence this population growth including age, race, diet, maternal colonization, as well as environmental exposures to xenobiotics and antibiotics [6]. Microbiota composition and complexity tend to change long GI in numbers and in species from proximal to distal tract and, at the same time, from epithelial surface and mucosal layer to intestinal lumen (Figure 1) [2]. All bacterial communities contain different gram-positive and gram-negative bacteria, whose main phyla are *Firmicutes, Bacteriodetes, Actinobacteria, Proteobacteria, Fusobacteria, Verrucomicrobia, Tenericutes and Lentisphaerae;* Furthermore main genera are: *Bacteroides, Clostridium, Faecalibacterium, Eubacterium, Ruminococcus, Peptococcus, Peptostreptococcus, Lactobacillus, Streptococcus, Streptomyces* and *Bifidobacterium* [1]. For their complexity it represented a challenge to phenotype all these populations in each individual and to compare them with others because factors mentioned above and some individual factors (stress, diet, travelling, drugs) can directly and rapidly influence their changes [1, 7]. So DNA-RNA sequencing methods have made possible to group clusters, known as Operational Taxonomic Units (OTUs), by genetic sequence similarities of specific taxonomic marker genes [8]. In this fashion microbiota and microbiome, often used as synonyms, express two different meanings: microbiota defines microbial communities, whereas microbiome defines their genetic information [9]. Alterations in gut bacteria compositions have been demonstrated to contribute and affect different diseases including metabolic disorders (obesity and Type 2 diabetes), inflammatory (inflammatory bowel disease-IBD), autoimmune disease (rheumatoid arthritis) allergy, irritable bowel syndrome (IBS), and cancer [10–14]. Cancer is one of the most leading cause of death in many western world countries, its incidence is related to many factors like population ageing, lifestyle choices as diets rich in red and processed meat or alcohol consumption or smoke, exposure to carcinogenesis or cancer suspect agents [15]. Many considerable progresses in figuring out its genetics and molecular/cell biological mechanism have been made for years [16]. Although understanding how environmental factors can have an impact on it and influences carcinogenesis is still an important challenge.

## GUT MICROBIOTA: HOMEOSTASIS AND DYSBIOSIS

Understanding functional gut microbiota could be important to clarify relationship with host immune system and host homeostasis. Mucosal immunity interacts (cross-talks), with the bacterial by environment establishing a symbiotic tolerance; which selects useful bacteria from pathogenic bacteria [17].

**Figure 2:**
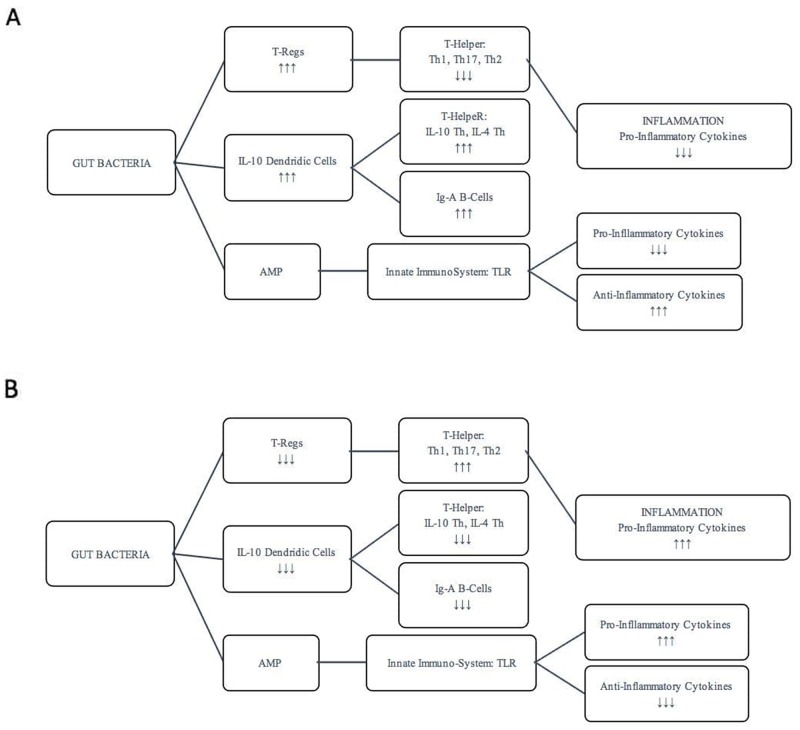
Regulation of intestinal immune system Regulation of intestinal immune system in pro-inflammatory **(A)** and anti-inflammatory conditions **(B).**

Aging, lifestyle and nutritional changes, stress, medications affect the composition of the microbiota with consequent alteration of inflammatory and pathophysiological states of the intestine and of numerous organs and tissues. A diet poor fiber and rich of fat determine alteration of Microbiome-associated metabolites, such as Vitamins B7 and B12 correlates with increased inflammatory state and frailty that caused a decreased gut mobility. Prolonged caloric restriction produces an increase *Lactobacillus,* while during dysbiosis there are a decrease of *Proeobacteria, Lentisphaerae, Bacteroides, Parabacteroides.* This conditions upgrade the serum levels of TNFα, IL8, IL1β and C-reactive protein [5]. Antigens derived from various species of bacteria (such as lipopolysaccharides or peptidoglycan) and from bacterial metabolism generate a response of immune cell types. The most activated cells are Transforming Growth Factor-β-producing-T regulatory lymphocytes of lamina propria and a special population of IL-10 producing Dendritic Cells (DCs) trough Toll-Like Receptors (TLRs) present on them [18]. Regulatory T-cells downregulate responses from pro-inflammatory T-helper cells such as T-helper type 1 (Th1), T-helper type 17 (Th17) and T-helper type 2 (Th2), while DCs are able to polarize naïve T-helper (Th0) lymphocytes to become regulatory cell producing IL4, IL10 and Tumor Growth Factor-β moreover also stimulate B-lymphocytes to produce commensal specific IgA (Figure 1, 2A) [19, 20]. These lymphocytes not only have a direct role dropping out inflammatory condition, but antimicrobial peptides (AMPs) secreted into mucus layer are critical molecules in controlling enteric microbiota by different ways. AMPs can directly kill pathogens, regulate enteric symbionts and immune response, moreover have chemotactic ability to recruit immune cells from monocyte and lymphocytic lineages [17, 21, 22]. Various external factors, such as antibiotic treatment, dietary changes, gastrointestinal pathogens and endogenous effects, may alter or deregulate the intestinal immune system, causing a potential dysfunction or a dramatic disruption to the microbial composition community [23, 24]. During dysbiosis several parameters like volume of water, ions concentrations, osmotic pressure and pH might be out of their physiological ranges of values of microbiota population [25]. This homeostatic inbalance might determine enormous growth of several bacteria and lead to chronic aggressive inflammation [23]. This chronic inflammation may damage the mucosa and immune system which gradually doesn’t crosstalk correctly with microbiota and doesn’t induce a correct immunological tolerance responsible for different possible diseases [26]. Have been shown that alterations in the immune-microbiotic-system relationship to contribute to the onset of diseases that have inflammatory pathogenesis (inflammatory bowel disease, colon rectal cancer, rheumatoid arthritis, type 1 diabetes, pulmonary disease, obesity, non-alcoholic fatty liver disease, atherosclerosis) [10–12, 27, 28]. In inflammatory conditions, intestinal immune system induces pro-inflammatory factors which down-regulate T-Regs cells and stimulate Th1 and Th17 to secrete pro- inflammatory cytokines and chemokines. Some of them, such as IFNγ and IL17A, are also considered to promote cell proliferation and/or inhibit apoptosis, while regulatory immunologic cells are down-set (Figure 2B) [26]. TLR (Toll-Like Receptors)-signaling pathway is useful to modulate microbial composition and set immune system-microbiota relationship; TLR may be considered as an interface among intestinal epithelial barrier, microbiota, and immune system Their pathway activation by pathogens is involved in the pathogenesis of several diseases [29]. As it has been demonstrated, TLR4 (Toll-Like Receptor 4), a cell surface receptor that senses bacterial lipopolysaccharides, increases proliferative and anti-apoptotic effects trough expression of hepatomitogen epiregulin in liver tumor development; while TLR2 (Toll-Like Receptor 2), which is expressed on the surface of T-Reg cell, has a pathway that can promotes immunologic tolerance and let the immune system to discriminate between commensal and pathogens bacteria [30, 31].

## GUT MICROBIOTA: EFFECTS ON TUMORIGENESIS

Gut microbiota has also been proved to plays an important role in cancer progression by modulating inflammation and influencing genomic stability of host cells through deregulation of different signals/pathways [32]. The chronic infectious agents to contribute to carcinogenesis approximately around of 18%. *Helicobacter pylori* and Hepatitis C virus, promote the development of cancer through epithelial injury and inflammation, contributes to carcinogenesis [32]. Gastric cancer can is caused by infection of a *Helicobacter pylori*, this pathogen bacteria is classified as a carcinogen by the International Agency for Research on Cancer (IARC). In Transgenic mice FVB/N insulin-gastrin (INS-GAS Mice) treatment with Helicobacter pylori accelerated atrophic gastritis and gastrointestinal intraepithelial neoplasia (GIN) respect to INS-GAS Mice naïve. Serum levels of cytokines and chemokines (IL1α, IL12p70, TNFα, IL17) in mice naïve are reduced respect to *H. pylori*–monoassociated. The up-regulation of interleukin-17 in the gastric tissue and serums associated with a Th17 response, results in gastric tumor pathology induced in *H. pylori* mono-associated Mouse INS-GAS [33, 34].

The Th17 and Stat3 are implicated, also in other types of carcinogenesis for example in colon rectal cancer (CRC). Enterotoxigenic Bacteroides fragilis (ETBF) secretes Bacteroides fragilis toxin (BFT) causes human inflammatory diarrhea but also asymptomatically colonizes a proportion of the human population. Mice colonized with ETBF, but not in non-toxigenic *B. fragilis* (NTBF), developed inflammation, hyperplasia and gastrointestinal intraepithelial neoplasia (GIN) foci. ETBF triggers colitis and strongly induces colonic tumors in multiple intestinal neoplasia (MIN) by Stat3 activation with colitis characterized by a selective T helper type 17 (Th17) response distributed between CD4+ T cell receptor-α/ β (TCR α/ β)^**+**^ and CD4/8-TCRγ/ δ^**+**^ T cells [35].

*Fusobacterium nucleatum*, which has been detected on the surface of over 50% of colon rectal adenomas, are able to promote intestinal inflammation within the tumor microenvironment in MIN; some members of *Escherichia coli* synthesize a genotoxin named colibactin, which is associated with DNA damage in CRC and IBD [36, 37].

In addition, gut bacteria can interact with food products and some inflammation-produced host metabolites increasing both the growth of some potential pathogen bacterial populations and the risk of DNA damage [38]. Some products of red meat and high processed food are metabolized by gut bacteria and some of their metabolites, such as hydrogen sulfide, are believed to damage DNA and to be mutagenic [39]. At the same time, bacteria could enhance the formation of other mutagenic factors like secondary bile acids such as deoxy- and lithocholic acids through the de-conjugation of biliary acids [40, 41]. Some of these products are hypotized to modify mucosal barrier allowing antigen penetration and increasing inflammatory factors production that boost genetic instability within the mucosal cells and increase production of factors useful for tumor development such as vascular endothelial growth factors (VEGF) and epidermal growth factor (EGF) [42]. On the other hand, some bacterial populations have been compelled to be protective against CRC thought metabolite production, induction of immune-tolerance, or an ability to outcompete pathogenic bacteria [43]. Although many organs, for example, the liver, do not show a known microbiome, they may be exposed to microorganism-associated molecular patterns (MAMPs) and bacterial metabolites through anatomical links with the gut [32].

*Helicobacter hepaticus* has been linked to activation of NF-kβ-regulated networks in liver cells for this pattern can promote hepatic cancer. The deregulation of NF-kβ, induces high production of nuclear β-catenin [44, 45]. Germ-free animals studies have revealed the evidence for liver tumor-promoting effects of the microbiota in carcinogen-induced cancers model. C3H/HeJ and C3H/HeOuJ mice, which carry respectively a non-functional and the wild type TLR4, exposed to combination of diethylnitrosamine (DEN) and the hepatotoxin carbon tetrachloride (CCl4), showed chronic injury, inflammation, fibrogenesis and CCl4-mediated increases of endotoxin levels, and thus shares several features with the microenvironment in which the majority of human HCCs arise. This suggests that inflammation and liver fibrosis linked to TLR, may induce an alteration of microenvironment that prepare the basis for HCC trough ligands that triggering the promotion of dependent TLR4 tumor from the intestinal bacterial microbial [30]. Recent studies suggest certain strains of bacteria can directly affect tumor development through the production of virulence factors like toxins or gene products either to direct manipulate inflammatory status of the tumor microenvironment or to influence the genomic stability of the host and to epigenetically regulate host gene expression [36].

## GUT MICROBIOTA: ANTIBIOTICS, PROBIOTICS AND PREBIOTICS’ INFLUENCE

Gut microbiota populations are modulated by some intake substances such as antibiotics, probiotics and prebiotics. Antibiotics are a group of drugs used for treatment and prevention of bacterial infections with different side effects that need to be faced. One of them is the death of commensal bacteria: these drugs may kill different types of bacteria which play a helpful role in the gut homeostasis [46].

The antibiotics are among the most important drugs used in human and animal medicine in the last 100 years. However, there are some negative aspects of antibiotic treatment that have been becoming more apparent in the last 20 years. **a)** Antibiotic abuse has selected for antibiotic resistance in many bacteria, and has made antibiotic treatments ineffective, claiming many lives, particularly those of compromised patients in hospital settings, like MRSA, VRE, or *Clostridium difficile*. **b)** It is getting more difficult to develop new antibiotics and there aren’t any indications that new antibiotics will be available in the next 10 years. **c)** A strong correlation has been observed between early antibiotic use and the development of allergic and autoimmune diseases [47, 48].

A clear example is the well documented lower incidence of gastric cancer induced by *H. pylori* in many western countries, where large treatments have been made despite of the more frequent esophageal cancer incidence [46]. On the other hand, in mouse model experiments different antibiotics treatments are applied to achieve a selective or a full depletion of several types of bacteria present in the gut [49].

The alteration of microbiome composition depends on the antibiotic class, dose, and period of exposure. There are also differences related to their pharmacological action or target bacteria; they are part of different classes and their antibacterial spectrum may be against main strains of gram-negative, gram-positive or anaerobic bacteria. A clear example is ampicillin, one of the most used, is often associated with some glycopeptide antibiotics and aminoglycosides to eradicate the highest possible numbers of bacteria; while metronidazole targets specifically anaerobic bacteria [50].

Macrolides are one of the most common antibiotics used around the world in children and adults. Many patients are taking macrolides for long periods of time due to a chronic infectious disease [45].

Currently, clarithromycin has been the first antibiotic selected for eradication of *H.pylori* with a decrease of *Actinobacteria* and *Firmicutes*, and an increase in *Bacteroides* and *Proteobacteria* [46]. In other studies has been reported the comparison between oral vancomycin and amoxicillin treatments in which vancomycin showed a major decrease in fecal microbiota diversity due to *Firmicutes* reduction and an increase in *Proteobacteria* [49]. Moreover penicillin reduced *Firmicutes,* but this reduction was minimal when compared to vancomycin; ciprofloxacin reduced *Firmicutes* and *Actinobacteria* (especially *Bifidobacterium*), and increased *Bacteroides* [50]. Another group showed a comparison between Ciprofloxacin and Clindamycin, with Ciprofloxacin decreasing *Bifidobacteria* and Clindamycin decreasing *Bifidobacteria* and *Lactobacilli*. However, no recovery of *Lactobacilli* was observed in the Clindamycin group [51–54].

Probiotics and prebiotics can hugely help to maintain healthy microbiota and restore a beneficial microbial composition. Probiotics like lactobacilli (*L. casei, L. rhamnosus*) are live bacteria that provide health benefits when consumed: they can be easily found in yogurt, cheese and other dairy foods [55]. Recent studies have shown that the lack of the presence of elafin can be among the contributory causes for the inflammatory bowel disease. The creation of engineered *Lactococcus lactis* which produces elafin, also known as peptidase inhibitor 3 or skin-derived antileukoprotease (SKALP), a protein encoded by the PI3 gene; this protein reduces cytokines production as it has been demonstrated in a mouse model of colitis [56]. Prebiotics are substances that enhance the growth or activity of gut microorganisms and are typically non-digestible fiber compounds, that pass undigested through the upper part of the gastrointestinal tract; some of them have been demonstrated to play an important role in cancer prevention, such as any phytoestrogens (resveratrol, entrolactone) present in certain berries which works as antioxidant and as down regulator of COX-2 mediated inflammation [57]. Fiber is one of the most well know prebiotics included in many fruits and vegetables; it’s fermented by gut bacteria into short-chain fatty acids that reach high levels in the lumen of colon [58]. Many studies have reported that butyrrate, one of short chain fatty acids produced by fiber fermentation, has tumor suppressive properties. It has been observed in some mice experiments that promotes the normal growth of colonic epithelial cells, and inhibits colon-rectal cancer cell growth due to Warburg effect [59]. Due to this effect colon rectal cancer cells up-regulate glucose intake and increase aerobic glycolysis as decrease oxidative metabolism; so in this way less butyrrate is metabolized in mitochondria and more is accumulated in nucleus where it acts as endogenous histone deacetylase inhibitor increasing transcription of cancer related genes important for cell proliferation apoptosis. So loss of butyrrate-producing populations in the gut could increase both inflammation and tumorigenesis [60].

Not only fiber but many other herbal supplements can be converted in potentially anti-cancer metabolites by gut microbiota as *American Ginseng*. Some studies have demonstrated that ginseng metabolites (CK, Reg3) can significantly attenuate colitis and colon carcinogenesis induced in mice models of colon cancer with Azoxymethane (AOM) followed by Dextran Sodium Sulfate (DSS) through gut cytokine levels reduction, restoration of endogenous metabolites levels and shift of microbiota population.

It was also reported that after *Ginseng* administration Gram-negative phyla (e.g., *Bacteroidales* and *Verrucomicrobia*), which possibly could promote tumorigenesis, decrease while Gram-positive phila (e.g., *Firmicutes*), which possibly could have anti-inflammatory and anti-tumorigenic, increase [61].

Recently *Ganoderma lucidum*, a medicinal mushroom used in traditional Chinese medicine, has been showed to reduce inflammation and maintain intestinal barrier integrity thereby decreasing metabolic endotoxemia in some mice models where dysbiosis was induced; at the same time its action is also considered to enhance growth of *Clostridum* XIVa and XVIII, which might have immunomodulatory properties such as induction of T-Reg cells, and prevent hepatic activation of TLR4 which is usually induced by metabolic endotoxemia [62]. As another study on melanoma and triple-negative breast cancer cells has demonstrated that *Ganoderma lucidum* inhibits the release of cytokines secreted by these cells under pro-inflammatory conditions due to administration of LPS, furthermore it has been revealed to decrease cancer cells viability and reduce cell migration according to a lower release of matrix metalloproteases [63]. Even association of probiotics and prebiotics has been demonstrated to have positive effects on reducing colon cancer models tumor incidence *in vivo* and to better stimulate NK cell activities as well as a mouse model study showed that saccharides like inulin, oligofructose, and dextran administered together with *Lactobacillus rhamnosus* or *Bifidobacterium lactis* more effectively enhanced NK cytotoxicity and cytokine production of spleen derived mononuclear cells [64].

## GUT MICROBIOTA: INTO CANCER

Several differences of microbiota compositions and bacterial/cells relations have been studying in numerous types of cancer. Different studies are focusing on bacterial density near mucous membrane which can change after dysregulation of tight junctions (such as E-cadherin and β-catenin) in epithelial tumors, while a recent study has reported that colorectal cancers associated with *Bacteroides fragilis* could be suppressed, not just by eradicating the microorganism but also by reducing inflammation [65, 66]. However, even if some pathogens are clearly capable of driving “*one microbe-one disease*” such as *Human papillomavirus* for cervical cancer, *Helicobacter pylori* considered a class 1 human carcinogen for gastric adenocarcinoma and some *Hepatis B virus/Hepatis C virus* for hepatocellular carcinoma, changes in microbiota are more evident when a combination of different shifts occurs rather than a single modest event (Table 1) [67, 68].

**Table 1:** Bacteria and Viruses directly involved in human cancer.

ORGANS/TISSUE	CANCER	BACTERIA/VIRUS RESPONSIBLE
**Oro-Pharynx**	Oro-Pharyngeal Carcinoma	Human Papilloma Virus
**Naso-Pharynx**	Naso-Pharyngeal Carcinoma	Epstein-Barr Virus
**Esophagus**	Esophageal Adenocarcinoma	Helicobacter pylori
**Stomach**	Gastric Adenoncarcinoma	Helicobacter pylori
**Stomach**	Gastric Lymphoma	Helicobacter pylori
**Liver**	Hepatocellular Carcinoma	Hepatitis-B Virus
		Hepatitis-C Virus
**Anogenital Tract**	Anogenital Carcinoma	Human Papilloma Virus
**Immune System Cells**	Lymphoma	Epstein Barr Virus
		Human Immunodeficiency Virus
**Bone Marrow**	Adult T-cell Leukemia	Human T-cell Lymphotropic Virus type 1
**Various**	Kaposi Sarcoma	Human Herpes Virus 8

**Figure 3:**
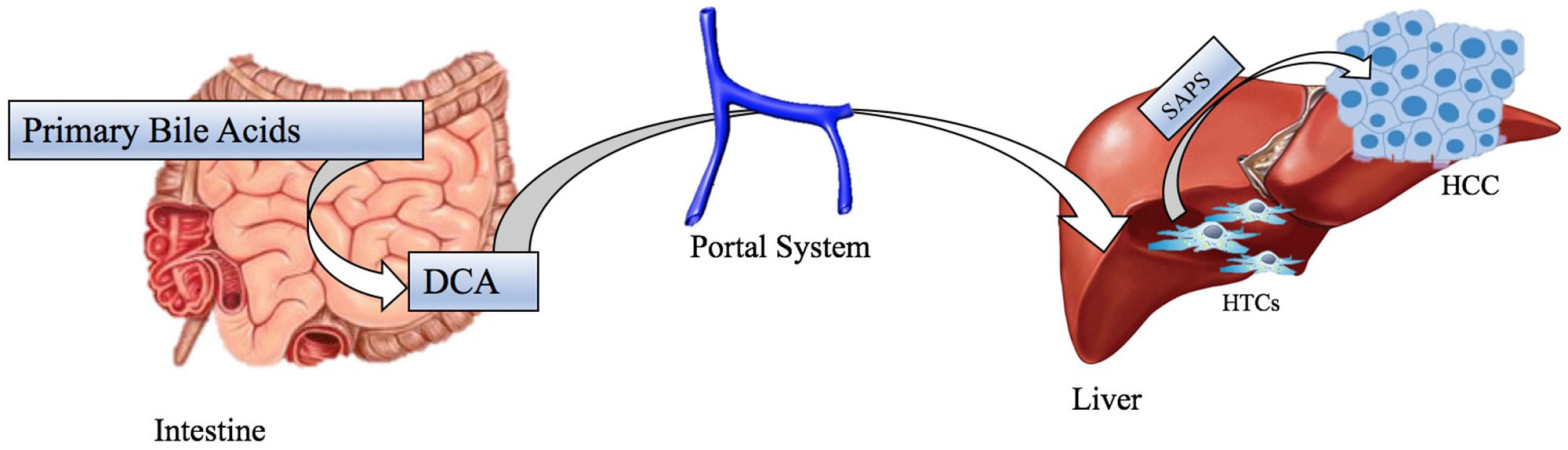
Etiology of hepatocellular carcinoma in model for obesity whit Gram Positive dysbiosis *Clostridium Clusters* convert bile acid in DCA, which arrive at liver by portal system. Elevated levels of DCA induces SASP in HSCs, which in turn, secretes various inflammatory and tumor-promoting factors in liver, that promote the making of HCC. Abbreviations: SASP: Senescence Associated Secretory Phenotype, DCA: DexyCholic Acid, HSCs: Hepatic Stellate Cells, HCC: HepatoCellular Carcinoma.

**Figure 4:**
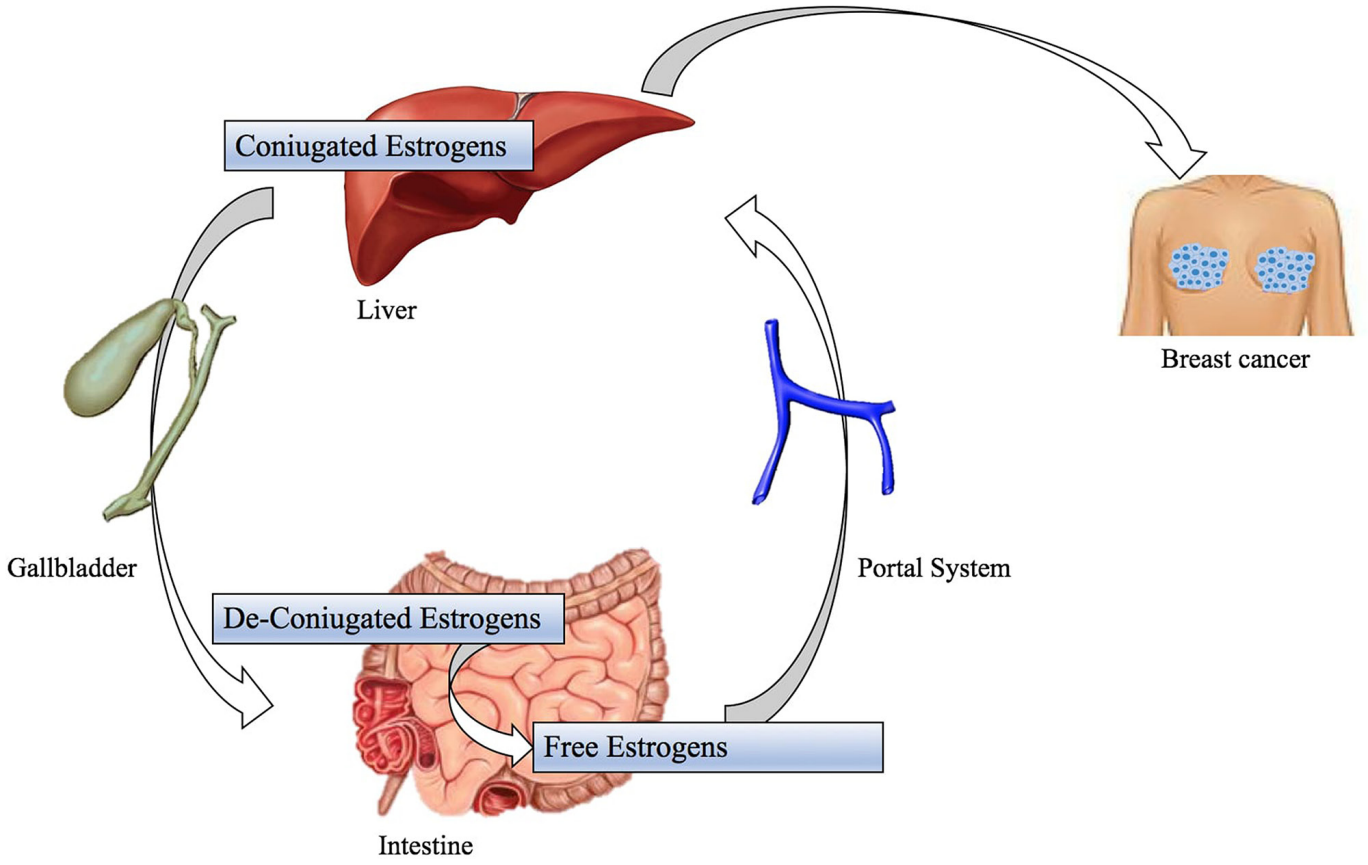
Possible mechanisms of gastrointestinal microbiome for the development of breast cancer Coniugated estrogens take place in liver. They are deconiugate by gut microbiota as free estrogens; these are reabsorbed through enterohepatic circulation. The reabsorption determine the elevated concentration of ‘‘estrogen-like substances’’ that induce the synthesis of ‘‘estrogen-inducible growth factors (estromedins)’’, which are polypeptides with carcinogenic potential with breast tropism.

Colorectal Carcinoma is one of the most diagnosed malignancies; many factors, as diet rich in processed meat and chronic inflammation of gastrointestinal tract, are significant for this cancer tumorigenesis and, as mentioned above, these ones can be associated with changes in gut microbiota population [69]. Recent studies have observed a shift in the composition of gut microbiota in both luminal microbiome and mucosa associated microbiota of patients with CRC compared to healthy controls but their results have disagreed in terms of the specific gut microbiome composition and profile related to CRC [70]. A Zakular et al.’s demonstrated that microbiome community wide changes promote tumorigenesis in the colon. In this study colitis-associated CRC model mice were treated with AOM followed by DSS and developed tumors. A group was traded only with DSS didn’t developed cancer, but had an important microbial community changes; while the synergic effects of the AOM/DSS model is necessary for the development of altered microbiome structure and tumorigenesis. In the mouse treated with AOM/DSS mice plus an antibiotic cocktail: it is observed that antibiotic intervention during inflammation reduced tumorigenesis. During the AOM/DSS tumor major shifts in bacterial populations from a wide range of taxonomic were observed and considered necessary for the development of the altered microbiome structure and tumorigenesis: tumor bearing mice showed enrichment in OTUs affiliated with *Bacteroides* (OTU 1) and *Turicibacter* (OUT 20) while there was a significant decrease in OTUs associated with members of genus *Prevotella* (OTU 4 -5) and the family of *Porphyromonadaceae* (OUT 7-12-15-22-31-48). In addition, germfree mice were conventionalized with either the healthy microbiome of untreated mice or the microbiome of tumor-bearing ones. Mice conventionalized with the microbiome of tumor bearing mice had a 2-fold increase in tumor burden relative to that of mice conventionalized with healthy microbiome, showing that alternated SPF gut microbiome transmitted to germfree mice could exacerbate colon tumorigenesis [71]. Another study by Vannucci et al. clarified gut microbiota role in carcinogenesis and anticancer immune response using germ free (GF) and conventional (CV) rats models. This demonstrated GF rats colon-rectal cancers were smaller than CV rats ones and tended to be singular entities rather than multiple tumors in different CV rats large bowels. This study also showed up cancer-resistant GF rats had a higher cytotoxic cells level in blood than healthy control and CV rat suggesting that lower antigenic challenge and absence of inflammation may enhance the capacity to develop more efficacious anti-cancer responses [72]. In this way, it was hypothesized that the continuous immunological activation to enclose the commensal flora assault together with bacterial antigens and drug-diet metabolites may considerably increase the spectrum of tolerated antigens [35]. So, a tolerant environment might override the capability to promptly respond to transformed cells as result [72]. The development of other neoplasms, such as Hepatocellular carcinoma, is induced by obesity, that is a clear significant risk factor for cancer, and its relation with gut microbiota is well documented even if molecular mechanism underlying obesity-associated cancer isn’t yet well understood [10]. So, some studies have demonstrated that leptin deficient obese mice (ob/ob) and fat diet fed wild-types mice have a higher risk to develop hepatocellular carcinoma than standard diet fed wild types [73]. One possible reason is an increased *Firmicutes phylum* bacteria population (Gram positive bacteria): these ones have a well-documented role in increasing calorie extraction and some of them, such as *Clostridium clusters* XI e XIVa (OTU1105), are able to convert bile acids into a secondary bile acid named deoxycholic acid (DCA) in the intestinal tract (Figure 3) [74]. The metabolism of estrogen takes places in the liver, where they are conjugated and excreted into the gastrointestinal lumen within bile; there they are de-conjugated by β-glucuronidase bacteria and then they are re-absorbed as free estrogens through enterohepatic circulation getting different organs like breast [75]. These metabolites are produced through estrogen metabolism by several bacteria included in *Clostridia* and *Ruminococcaceae* families which could be over-expressed during dysbiosis. In addition, other estrogen-like metabolites can be also produced by oxidative and reductive reactions in gut and induce synthesis of estrogen-inducible growth factors (estromedin), which might have a carcinogenic potential (Figure 4) [76]. Otherwise, even relationship between diet and risk of breast cancer has been examined: phytoestrogens, plant-derived xeno-estrogens, can have anti-estrogenic effects and reduce risk of breast cancer as it has been showed in some epidemiologic studies [77]. An example is enterolactone which is produced by microbiome fermentation of dietary lignan and has been reported to be a potential anti-proliferative agent for breast cancer [78].

## GUT MICROBIOTA: IN CHEMOTHERAPY AND IMMUNOTHERAPY

Gut microbiota takes part not only in cancer tumorigenesis but in cancer prevention and chemotherapy efficacy. As recent studies have reported, certain bacteria may improve effect of some traditional anti-neoplastic drugs and immunotherapy drugs. Cyclophosphamide (CTK) is an alkylating drug used in combination with other anti-cancer molecules whose effects has been demonstrated to be modulated by commensal bacteria. A study based on GF mice and specific pathogen free mice (SPF) evidenced CTK therapeutic efficacy has been improved by some gram-positive bacteria: after CTK induces a disrupt of intestinal epithelial barrier, bacteria and their metabolites translocate through it and stimulate systemically Th17 and Th1 cells to a straight anti-tumor immune response [79, 80]. In addiction oxaliplatin and cisplatin and CpG oligonucleotides (ODN) have also been studied to have an anti-tumor response based on gut-microbiota interaction: in mice developing subcutaneous lymphomas treated with antibiotics, intestinal bacteria may prime myeloid cells to release reactive-oxygen species (ROS) which enhance oxaliplatin anticancer damages [81]. *Candida* species can drive myeloid cells to infiltrate tumor sites and increase production of pro-inflammatory cytokines as commensal fungal that its overgrowth, this phenomena is observed following antibiotics-induced gut dysbiosis and it has been shown to result in increased prostaglandin E2 plasma concentration and M2-macrophage polarization in the lung leading to heightened allergic airways inflammation [82]. Furthermore also tumor necrosis factor (TNF), by myeloid cells which ODN stimulates through TLR9 binding. All these conditions are responsible for a rapid hemorrhagic necrosis in the treated tumors [83]. Recent studies furthermore proved that in melanoma patients responders for anti-PD1 immunotherapy showed a high diversity and abundance of *Ruminococcaceae*/ *Faecalibacterium* with increased antigen presentation and effector T cell in the periphery and in the tumor microenvironment [84]. Intestinal *Bacteroides* species such as *Bacteroides thetaiotaomicron* and *Bacteroides fragilis* have been confirmed to improve immunity against cancer through antibodies directed against lymphocyte-associated antigen-4 (CTLA-4) by modulating dendritic cells. This treatment induces a mucosal damage and a microbial translocation of *Bacteroides*, which are necessary to activate immune system cells and create a more suitable anti-tumor environment [85]. Althouth the microbiota shows benefical effects for the organism that are altered for instance when are administrated wide range antibiotics inducing a dysbiosis sometimes microbiota can also influence occurrence of adverse side effects; one example is the administration of irinotecan (CPT-11), toposimerase I inibitor. After intravenous administration CTP-11 is transformed in SN38 produced by carboxylesterases and is glucuronidated in the liver by uridine diphosphate (UDP)–glucuronosyltransferase enzymes to form inactive SN-38G which is excreted via the biliary ducts into the gastrointestinal (GI) tract; When it reaches the bowel, it is again activated by some commensal bacteria inducing a dose-limiting diarrhea through epithelial barrier damage [86].

## CONCLUSIONS

Gut microbiome is an extremely complex community and its shifts, called dysbiosis, is related to several factors whose mechanism isn’t clarified yet. One of them mechanism, which is even important in cancers development, is the relationship between inflammatory cytokines bacteria and immune system. In this context immunity sets a deep connection show an important crosstalk with gut microbiome; bacterial signals modulate immunity response and at the same time alert immune cells, to hit the right target.

The gut microbiome have local and systemic effects on cancer disease. In particular, influence the pathogenesis of stomach cancer by *Helicobacter pylori*, Colorectal Carcinoma by *Escherichia coli*, *Fusobacterium spp*. and enterotoxigenic *Bacteroides fragilis*, and Bladder Carcinoma by *Salmonella enterica typhi*. Furthermore it’s implicate in others neoplastic pathology: lymphoma, prostate cancer, breast carcinoma, sarcoma, pancreatic cancer, ovarian cancer, hepatocellular carcinoma, through different and various mechanisms, that involving the innate and adaptive immune cell migration, endocrine and neural pathways, and translocation of bacteria or bacterial products and toxins, and modulation of the systemic inflammatory tone ant the oxidative stress.

This connection is responsible for homeostasis control and tumor microenvironment regulation. However, despite some exceptions such as some single bacteria strains responsible for driving directly some types of tumors, different commensal bacteria groups have been demonstrated to modulate locally carcinogenesis and contribute to cancer pathogenesis in an altered homeostasis environment, even if mechanism behind them is still an area of intense research. Otherwise, their effect on carcinogenesis has been also considered systemically: gut microbiota shifts have also been included in one of the different causes for breast cancer but their indirect effect could be associated to other neoplasms where this association is still unknown.

In cancer therapy microbiome has been also achieving importance for modulating chemotherapy drugs efficacy and side-effects. Several studies have been focusing on the chance to enhance drugs effect and to reduce side effects hiring new possible approaches by targeting the microbiota.

In this fashion the microbiota is thought as an good and active strategy to fight cancer: some other functions of commensal microbes might be beneficial against cancer trough their interactions with immune system or production of metabolites. So, understanding how bacteria shift and its immune system relationship are the keys to set gut microbiome as a holistic hub point for cancer development and, at the same time, improve clinical and therapeutic approach.

In the near future, it won’t be a utopia if gut microbiota can be used as a biomarker to differentiate healthy and tumor patients while prebiotics and/or probiotics can be used either as preventive care to restore the healthy gut microbiota and to stop cancer development or be administered concurrently within chemotherapy protocols in oncological hospitals.
